# COVID-19 and social services in Spain

**DOI:** 10.1371/journal.pone.0241538

**Published:** 2020-11-18

**Authors:** Rocío Muñoz-Moreno, Alfonso Chaves-Montero, Aleix Morilla-Luchena, Octavio Vázquez-Aguado

**Affiliations:** Department of Sociology, Social Work and Public Health, University of Huelva, Huelva, Spain; Faculty of Science, Ain Shams University (ASU), EGYPT

## Abstract

During the state of alarm declared in Spain by COVID-19 due to the pandemic, the country's authorities declared Social Services and their workers to be essential, considering that the activity of these professionals with the vulnerable population was crucial and that services should continue to be provided to guarantee the well-being of users in this exceptionally serious situation. This article analyzes the impact that the COVID-19 and the state of alarm has had on Spanish social service professionals. An ad hoc questionnaire was used, administered on-line, individually, voluntarily and anonymously to 560 professionals working in social services, both in the public and private sectors, based throughout Spain. This questionnaire has five different parts: socio-demographic profiling, impact that the health crisis has had on the practice of professional functions, degree of knowledge of the measures imposed to guarantee the protection and safety of professionals and users, impact that it has had on the professional and personal development of social services professionals and, the fifth and last part, degree of adaptation of the measures aimed at the care of the vulnerable population. These results are discussed based on the situation in which professionals working in this sector find themselves in the face of the changes they are experiencing in the development of their work, and we are able to determine the profile of the workers who have felt most affected by the situation, with the consequent and foreseeable mental and emotional affectation that this implies. These professionals tend to value more negatively the set of measures developed to mitigate the impact of COVID-19 on Spanish social services.

## Introduction

Since COVID-19 began to spread in China, it had been 5 months since the first case was detected until the time this article was written [1]. The speed of its expansion is only comparable to the impact of its health, social and economic consequences [2]. From Southeast Asia to Europe, the entire American continent and, apparently to a lesser extent perhaps because there is less information about it, Africa, all countries have been affected, although to a different degree. However, although the spread of the virus knows no borders and does not distinguish between people, it is no less true that the response capacity of countries and the resources of all kinds that people possess create enormous differences when it comes to limiting the socio-economic impact of this new disease [3].

Its spread has been dizzying. As of May 18, 2020, according to the daily report from John Hopkins University [4], more than 4.8 million people are infected worldwide, and more than 318,000 have died, with a particularly strong impact in Europe (Italy, Spain, France) and America (United States).

In the case of Spain, the first isolated cases occurred at the end of January 2020. These were tourists who were treated in the Canary or Balearic Islands. However, the spread of the virus was exponential, so much so that the government decreed a state of alarm among the harshest in Europe that confined all citizens to their homes, allowing only sporadic outings for shopping, going to the doctor or handle some banking transaction. All socio-economic activities were suspended, except for essential activities concerning the production, distribution and marketing of basic necessities. Among them, the activity of Spanish social services. This set of measures has had a great impact on the entire population, considerably increasing unemployment, contracting economic activity as never before and making the living conditions of millions of people, including the users of social services, even more precarious.

At the beginning of the state of alarm in Spain, social services were not considered an essential service. This led to the cessation of almost all the activity that had been taking place. Social intervention projects were cancelled, home help for the elderly and dependents was limited, and thousands of aids that contributed to the achievement of a minimum level of welfare for large sections of the Spanish population were no longer provided. The authorities quickly understood the impact of limited social services and declared them to be essential services. Thus, on 26 March 2020, 12 days after declaring the state of emergency in the country, the Spanish authorities, by order SND/295/2020, declared the social services and their workers to be essential, which allowed these services to continue, although subject to the state of emergency.

In this context, action in the social services has sought to ensure continuity, under exceptional conditions. The first measures were aimed at ensuring the provision of home help, which has consisted of: reconfiguring the care content, modifying the actions according to priorities, combining them with other services (tele-assistance, home help, etc.), as well as in connection with the personnel of the administration and of the companies providing services, all this in order to ensure the provision of the service in accordance with the safety conditions of both workers and users. These guidelines for action have also affected homeless people, with the aim of ensuring the provision of social services and establishing measures to achieve their confinement. Recommendations were also issued to mitigate the impact of COVID-19 and the state of alarm in segregated settlements and highly vulnerable neighborhoods. These recommendations have been extended to programs for the protection of children and adolescents, volunteering and intervention in primary care social services.

In this article we will analyze the perception that Spanish social service professionals have had of both the impact of COVID-19 and the state of alarm on the living conditions of the vulnerable population and the measures that have been taken to mitigate its effects. We will present results concerning four main questions: how they think it has affected the social services, how have the professionals felt during the first weeks of the state of alarm, what knowledge do they have of the protective measures put in place in the workplaces and, finally, how has it impacted on the living conditions of the vulnerable groups they work with. We will show the overall results and will stop at the assessment made by the professionals most affected by this situation.

## Materials and methods

### Instrument

The results shown in this paper are based on a questionnaire administered to professionals working in social services, both in the public and private sectors, based throughout Spain. A total of 560 professionals from the sector participated. The field work was carried out from April 1st to April 19th.

The questionnaire administered consists of five separate parts: a first part concerning the socio-demographic characteristics and the professional situation; a second section, of 16 items, aimed at the assessment of the professionals on the impact that the current situation of health crisis and state of alarm has generated in the usual practice of the social services; a third section which is to do witch the knowledge of the protection measures that have been carried out in the different organizations (8 items); a fourth section aimed at analyzing the professional and personal situation with the current situation (13 items); and a fifth and final section concerning the adequacy of measures to address the vulnerable population (24 items).

You can consult the complete questionnaire with the questions translated into English and the items that are part of each of these sections in Appendix 1. This work complies with the requirements of the Ethics Committee of the Vice-Rector's Office for Research of the University of Huelva. As it is not experimental research, no specific authorisation document is required.

### Analysis strategy

In order to facilitate the analysis of the results, factorial analyses have been carried out, with varimax rotation, of sections two, four and five (measured on a likert scale) to reduce the number of items and work with this information in an aggregate manner. The resulting factors have been transformed into a 0–100 scale to facilitate their presentation.

Given the health emergency caused by the pandemic and the confined situation in which we found ourselves during the period in which the fieldwork is being carried out (from 1 to 19 April 2020), the decision was taken to develop an online tool through Google Forms, which would facilitate the collection of information from social service professionals. The global pandemic situation has limited the traditional conditions of field work in social research and has forced the academic world to rethink and introduce new methodologies that allow access with greater guarantees to the population under study.

In addition to the impossibility of accessing the respondents during the alarm state, the administration of this online questionnaire has other important qualities, such as the reduction of costs, the ease of access to it through any electronic device, as well as the possibility of reaching a larger target population in a shorter period of time.

Different authors endorse the use of this information collection tool, as it has proven to be methodologically practical, suitable and especially useful for obtaining a satisfactory statistical product [5, 6]. It has also been applied in other studies [7].

In terms of how to access the target population, a multiple invitation protocol is applied [8, 9]. Through a snowball process. The questionnaire has in turn been disseminated through social networks and shared by different professional associations, thus ensuring that the instrument reaches the greatest possible number of professionals within this field. Although a stratified sampling by quotas has not been established, given the difficulty of obtaining a detailed census of social service professionals in Spain, and which was also interested in reaching the greatest possible number of responses, all the Spanish provinces are represented.

In this paper we will focus, in addition to the general data, on the presentation of the results concerning a factor that we have called "Feeling overwhelmed by the situation", within the dimension "Assessment of the professional/personal situation", which in turn is composed of five items:

1. I have often felt like crying these days.
2. Throughout these days, argues with colleagues from social services have increased.
3. During these weeks, I have worked many more hours beyond my working hours.
4. There have been times when I have felt overwhelmed by the situation.
5. I have often felt helpless these days.

### Participants

The participants in the study through the self-administered questionnaire, are professionals who have been developing their activity in the field of Social Services with an average of 13 years in the service in which they carry out their work at present. Their socio-labor characteristics are reflected in the following table (Table 1):

**Table 1:** Socio-demographic characteristics.

		Frequency	Percentage
**Gender**	**Woman**	466	83,4
	**Man**	93	16,6
**Degree**	**Social Work**	410	73
	**Other qualifications**	150	26.
**Level of studies**	**Secondary**	12	2,1
	**University students**	395	70,5
	**Master**	133	23,8
	**PhD**	20	3,6
**Marital status**	**Married / Domestic partner**	287	51,3
	**Divorced / Separated**	51	9,1
	**Single**	216	38,6
	**Widower**	6	1,1
**Working status**	**Full-time worker**	491	87,7
	**Part-time worker**	59	10,5
	**Volunteering**	10	1,8
**Type of organization**	**Concerted (Public-private partnership)**	43	7,7
	**Private**	134	23,9
	**Public**	381	68,0

Source: author’s own.

## Results

In relation to the results, we will first present an approximation of the global data through the analysis of the average scores of the different factors obtained, as well as the correlations between these and the impact of COVID-19 and the state of alarm in social services. Secondly, the variables that have influenced the fact that social service professionals have felt overwhelmed by the situation will be addressed more comprehensively.

The following table (Table 2) shows the factors that have been obtained from the different dimensions of the questionnaire, as well as the number of items that compose them and their internal consistency, measured through the Crombach alpha.

**Table 2:** Factorial analysis.

Questionnaire dimensions	Factors obtained	N° of items that make up the package	Crombach's Alpha (internal consistency)
**Assessment of the impact of COVID-19 on professional development**	1. Degree of preparedness and effectiveness of measures implemented.	8	,822
	2. Degree of affectation of functions by state of alarm.	4	,795
	3. Efficiency Teleworking.	3	,722
**Assessment of professional/personal situation**	1. Feeling overwhelmed by the situation	5	,777
	2. Appropriate resources and measures to address the situation.	6	,740
	3. Teleworking.	2	,726
**Assessment of the degree of appropriateness of the measures aimed at the vulnerable population**	1. Worsening conditions of vulnerable population in a state of alarm.	9	,947
	2. Degree of adequacy of measures to assist the vulnerable population.	8	,862
	3. Effectiveness of collaboration and socio-health monitoring.	4	,774

Source: author’s own.

### General perception of the situation

In Table 3 we can see the average scores, on a scale of 0–100, of the different dimensions that make up the questionnaire, grouped into factors.

**Table 3:** Average scores obtained in the dimensions of the questionnaire.

	N	Mean	Standard deviation
**Degree of preparedness and effectiveness measures implemented.**	465	45,50	23,05
**Degree of affectation of functions by state of alarm.**	365	79,06	22,41
**Efficiency Teleworking.**	501	42,00	30,32
**Feeling overwhelmed by the situation.**	460	53,45	25,73
**Resources and appropriate measures to address the situation.**	367	59,53	23,64
**Teleworking Conditions.**	414	56,61	37,54
**Worsening conditions of vulnerable population in a state of alarm.**	313	70,17	29,42
**Degree of adequacy of measures to assist the vulnerable population.**	256	41,85	22,59
**Effectiveness of collaboration and socio-health monitoring.**	358	58,71	24,77

Source: author’s own.

In general, the assessment of all the measures developed is low. In the region of 50% or even less. There are only two of them that are above 50%, and they are those that most emphasize the negative aspects of the impact of the disease on social services: almost 80% of social service professionals estimate that their functions have been altered by the decree of the state of alarm that confined the population to homes. On the other hand, 70% of them consider that the living conditions of the vulnerable population will worsen because they believe that the measures being implemented for their protection are not adequate.

In this general assessment there are some differences that are worth highlighting according to the socio-demographic characteristics of the people participating in the sample.

Social care professionals perceive themselves to a greater degree to be overwhelmed by the situation compared to professionals with other qualifications who are part of the social service system.

On the one hand, persons who have children value being teleworking more than the rest and, in turn, they perceive greater effectiveness in having implemented this modality of work. However, people with children appear to be more overwhelmed by the situation and perceive a greater degree of worsening of the conditions of the vulnerable population with the state of alert.

In terms of age, it is the younger population groups (between 26 and 46 years of age) that show a greater degree of feeling overwhelmed by the situation and a greater worsening of the conditions of the vulnerable population with the state of alarm, although they also value more positively the degree of adequacy of the measures of attention to the vulnerable population and the fact that they have had adequate resources and measures to deal with the situation. It is the older people (over 60 years) who perceive a greater degree of affectation of their functions by the state of alarm. However, it is also the group that most values the degree of preparation and effectiveness of the measures implemented.

Finally, it is the women who say they feel most overwhelmed by the situation. In the rest of the factors analyzed, the highest average scores are found in men, with a notable difference in average scores compared to men who perceive to a greater degree (+7.94) that they have had adequate resources and measures to deal with the situation.

However, if we associate the factor concerning the degree to which professional functions are affected by the state of alarm with the other factors that are part of the different dimensions of the questionnaire (Table 4), the data show that there are significant correlations at the level of p < 0.01 between this factor and feeling overwhelmed by the situation (.399**), as well as with the worsening of the conditions of the vulnerable population (.380**). In other words, the social service professionals who perceive the situation to be worse are those who consider that it affects both the fulfilment of their functions and the worsening of living conditions of vulnerable populations.

**Table 4:** Correlations between the degree to which the functions are affected by the state of alarm and the rest of the factors.

	F2 scale P2. Degree of affectation of functions by state of alarm		
	Pearson Correlation	Sig. (bilateral)	N
**Degree of preparedness and effectiveness measures implemented.**	,181**	,001	316
**Efficiency Teleworking.**	,158**	,003	344
**Worsening conditions of the vulnerable population in a state of alarm.**	,399**	,000	222
**Effectiveness of collaboration and socio-health monitoring.**	,311**	,000	252
**Feeling overwhelmed by the situation.**	,380**	,000	328
**Resources and appropriate measures to address the situation.**	,332**	,000	269
**Teleworking**	,277**	,000	292

Source: author’s own.

There is also a positive correlation between this factor and the perception that resources and measures are adequate to deal with the situation during the state of alert (.332**) as well as with teleworking (.277**). It is estimated that these measures are adequate but not sufficient because everyone has not been able to access teleworking due to lack of resources, means, family situation or because they are not able to carry out all the functions that are carried out in person telematically.

### Perception of the situation by people who have felt overwhelmed in their professional development during the pandemic: In relation to the employment situation of professionals

It can be seen that the people who have felt most burdened by the situation are among the voluntary sector, as well as those who work full-time, albeit at a considerable distance. With regard to the time they have been working, we found that the professionals who have been working for less time in the same service or organization are those who say that they feel more overwhelmed by the situation, as opposed to those who have been working longer and have more experience in carrying out their work. If we put this fact in relation to the position held, we find that the people who carry out functions of management, coordination and responsibility of the team are those who show to a greater degree that they feel overwhelmed by the situation. Finally, considering the type of organization in which they carry out their work, it can be seen that the professionals in the concerted organizations (public-private partnership) show to a greater degree that they feel overwhelmed by the situation. We can see these characteristics in the following table (Table 5).

**Table 5:** Workplace characteristics of people who feel overwhelmed by the situation.

		Scale F1 P4 Feeling overwhelmed by the situation scale 0–100
**Working status**	Volunteering	**67,50**
	Part-time work	48,37
	Full-time job	53,70
**Time working in the same service /organization**	0–10 years working	53,96
	11–22 years working	54,31
	23–33 years working	50,90
	34–44 years working	45,38
**Position held**	Management functions, coordination, team responsibility	**60,43**
	Program Manager	54,86
	Technician	50,94
	Administrative work	55,00
	Other functions	57,67
**Type of organization you work for**	Private	50,58
	Concerted (Public-private partnership)	**60,41**
	Public	53,66

Source: author’s own.

### In relation to the perception of the degree of adequacy of the measures aimed at the vulnerable population

The following table (Table 6) shows the significant correlations obtained with the disaggregated items of section five (the degree of adequacy of the measures aimed at the vulnerable population) and the factor 1 "Feeling overwhelmed" obtained from section four (professional and personal situation).

**Table 6:** Correlations between feeling overwhelmed and the measures developed to attend to the vulnerable population.

	Scale F1 P4 Feeling overwhelmed by the situation		
	Pearson Correlation	Sig. (bilateral)	N
**The measures taken for the social and health monitoring of this population have allowed adequate attention to be given to it.**	,119*	,020	382
**I believe that there are unmet vital needs.**	,239**	,000	415
**The paralysis of social intervention projects (accompaniment) in relation to socio-labor inclusion is causing a standstill in access to potential jobs for the vulnerable population.**	,138**	,006	390
**The paralysis of administrative procedures for access to the Minimum Income of Insertion is aggravating the living conditions of the most vulnerable population.**	,201**	,000	390
**The suspension of Day Care Centers and Home Help Services that are not considered Minimum Services generates a problem of conciliation in the carers of dependent people and an overload in their care tasks.**	,178**	,000	410
**Confinement has severely affected people with mental health and mental illness and their families.**	,205**	,000	402
**Confinement is seriously affecting women victims of gender-based violence and their children.**	,185**	,000	393
**Most Social Services users do not have access to telematic procedures, and telephone assistance is sometimes not sufficient.**	,192**	,000	420
**The state of alarm and the health alert has made people vulnerable who, until now, were only in precarious conditions.**	,146**	,003	416
**In general, I believe that, once the state of alarm and the health alert is over, the living conditions of the vulnerable population will have worsened.**	,132**	,007	423

Source: author’s own.

As can be seen, the highest and most significant correlations with feeling overwhelmed by the situation are produced by the perception that there are vital needs that are not covered in the care of these groups (,239**); the paralysis of social intervention projects (accompaniment) in relation to socio-labor inclusion are hindering the access to potential jobs for the vulnerable population, along with the lack of employment and the future economic situation that is not very favorable (,138**); with the paralysis of administrative procedures, it is not possible to perceive and access the Minimum Social Insertion Income is aggravating the living conditions of the most vulnerable population (the elderly, minors, migrants, etc.)), which is the group that is punishing this health crisis the most (.201**); the suspension of the Day Centers and Home Help Services that are not considered essential services generates a problem of conciliation in the carers of dependent persons and an overload in their care tasks, without knowing in many cases how to deal with the situation due to a lack of resources, means, information and protective materials for the care of these dependents (.178**); confinement has seriously affected persons with mental health problems and mental illness and their families (.205**); confinement is affecting very seriously women victims of gender violence and their children, in which in most cases they live with their aggressor (.185**); with the fact that most of the persons using Social Services do not have access to telematic procedures, and telephone attention is sometimes not sufficient (.192**); the state of alarm and health alert has made the population vulnerable, which up until now was only in precarious conditions (.146**); and with the perception that once the state of alarm and health alert has passed, the living conditions of the vulnerable population will have worsened (.132**).

### In relation to the perception of the degree to which their functions have been affected by the state of alarm

We will now discuss the significant correlations that exist at a statistical level between the variables that make up the factor "I feel overwhelmed by the situation", in a way that is consistent with the set of questions raised in the survey regarding the assessment made by professionals of the impact that COVID-19 and the state of alarm have had on the performance of their functions (Table 7). It should be remembered that this factor is made up of 5 variables.

**Table 7:** Correlations between feeling overwhelmed by the situation and affecting professional functions.

	I've often felt like crying these days.	Throughout these days, discussions with colleagues from social services have increased.	During these weeks, I have worked many more hours beyond my working hours.	It can be said that there have been times when I have felt overwhelmed by the situation.	I have often felt powerless these days.
The work that we develop from the social services has been very affected by the appearance of the COVID-19 and the state of alarm.	,090*	,115**	,208**	,190**	,113*
I think that in the service where I work we were sufficiently prepared for a situation like this.	-,106*	--	--	--	-,146**
The response offered by the social services as a system has been adequate to the situation created.	--	--	--	--	-,092*
The coordination between institutions to organize the response of social services as a system to COVID-19 has been satisfactory.	--	-,163**	--	--	--
As professionals, we have had clear and concrete instructions on how to act in this situation	--	-,176**	--	--	-,121**
In my job, teleworking has allowed me to develop my professional work normally.	--	-,164**	,207**	--	--
I have had sufficient means to telework during the development of the crisis.	-,109*	-,207**	,149**	--	--
I have had the necessary training and instructions to be able to carry out my work telematically or not.	-,128**	-,259**	,124**	--	--
The declaration of social service professionals as essential seems to me to be correct	--	--	,229**	,177**	,152**
In general, it can be said that the social services system is overwhelmed by this situation.	,176**	--	,244**	,294**	,346**
The available human resources are sufficient to develop our services during the state of alarm	-,157**	-,120**	--	-,145**	-,210**
My usual functions have been altered during the crisis period	,152**	--	,170**	,254**	,263**
The implementation of the new measures derived from the state of alarm has been done effectively and efficiently	--	-,088*	--	--	--

Source: author’s own.

The first of the factor items, often in these days I have felt like crying, has a positive relationship with the perception of the degree to which work has been affected by the outbreak of the epidemic and the state of alarm (.090*); to the degree to which social services are perceived to have been overwhelmed by the situation (.176**) and to the degree to which normal functions have been altered during the crisis period (.152**).

On the contrary, negative correlations are produced, which have to do with the perception of the degree of preparation for the situation of the services in which the work is carried out (-.106*); with the fact of having sufficient means to carry out teleworking during the crisis period (-.109*); with the availability of information and instructions necessary to carry out the work telematically or not in person (-.128**); and finally with the perception that the human resources available are sufficient to carry out the service during the state of alarm (-.157**).

With respect to the second item (increasing argues with social service colleagues), it is also positively associated to the perception that the development of the work has been affected by the outbreak of the epidemic and the state of alarm (,115**). And it correlates negatively with the following aspects: "The coordination between institutions to organize the response of social services as a system to COVID-19 has been satisfactory" (-.163**); "As professionals, we have had clear and concrete instructions on how to act in this situation" (-.176**); "In my job, teleworking has allowed me to do my professional work normally" (-.164**); I have had enough means to telework during the crisis period" (-.207**); "I have had the necessary training and instructions to be able to carry out my work in a telematic or non-presential way" (-.259**); "The human resources available are sufficient to perform our services during the state of alarm" (-.120**) and with "The implementation of the new measures derived from the state of alarm has been done in an effective and efficient way" (-.088*).

As regards the third item, which has to do with the fact of spending more time than is established in the usual working hours, this is positively associated to the degree to which it is perceived that the work carried out by Social Services has been affected by the outbreak of the epidemic and the state of alarm (.208**), with the degree to which it is perceived that their usual work has been able to be carried out normally by means of teleworking (.207**); having sufficient means to telework during the crisis period (.149**); having the training and/or instructions necessary to carry out teleworking (.124**); the degree of agreement with the declaration of social services as essential (.229**); the perception that the Social Services system is overwhelmed by the situation (.244**); and the degree to which it is perceived that the usual functions have been altered (.170**).

The fourth point, concerning the fact of having felt overwhelmed by the situation, has to do with the following aspects: "The work that we perform from the social services has been very affected by the appearance of the COVID-19 and the state of alarm" (,190**); "The declaration of the professionals of the social services as essential seems to me to be right" (,177**); "In general, it can be said that the social services system is overwhelmed by this situation" (,294**); "The human resources available are sufficient to carry out our services during the state of alarm" (,145**); and "My usual functions have been altered during the crisis period" (,254**).

As for the fifth and last item that makes up this synthetic index, concerning to the appearance of a feeling of powerlessness with regard to the situation generated, this is positively associated to the degree to which the declaration of social services as essential services is perceived to have been correct (,152**), as well as to the degree to which the situation of social services is perceived to have been overwhelmed (,346**). However, there are negative correlations with the perception that the service in which they are working is sufficiently prepared for the situation (-,146**); with the perception that the response offered by the social services is correct (-,092*); and the degree to which they are perceived to have received clear and concrete instructions to act on that situation (-,121**).

## Discussion

The profile of the person who has felt overwhelmed by the health crisis is: a professional woman in Social Work, middle-aged (35–46 years old) and without dependent children.

The professionals who work in the Social Services, as well as the people belonging to the vulnerable groups they attend to, are being affected by the coronavirus pandemic, an unprecedented situation in professional practice and personal development. It is a situation of great impact and special vulnerability, as has been seen in the significant number of participants who say they have been overwhelmed by the situation, despite being professionals used to working in situations of risk and stress.

This crisis requires an exceptional effort of self-regulation to be able to be and give the best to each person who comes to the social services while the professionals must adapt to the seriousness of the situation, the loneliness that characterizes it and the isolation, even suffering the loss of users (especially those social professionals who are in the front line of intervention: residences for the elderly and hospitals) [10]. Precisely, among the sectors of population more taken care of by the social workers in Spain we can identify the people in situation of dependency (9.5%) and the old people (8.6%) [11].

Confirming the complexity of current public health scenarios caused by COVID-19, recent studies show the impact of these new demands for care on the physical and mental disposition of health workers, especially those working in direct care: doctors and nurses. Complaints of symptoms associated to distress, insomnia, depression and anxiety have been frequent among the categories mentioned. In addition, there is a negative impact on work, such as reduced satisfaction and productivity [12].

A parallel can be drawn between health professionals and those who are part of the Social Services system with regard to stressors arising from the care of users or patients: proximity to daily suffering, emotional involvement and added difficulty in maintaining therapeutic distance, a feeling of lack of resources for care (material and human)… [13], to which is added the crisis situation itself derived from COVID-19 and affecting to a greater or lesser degree all professional sectors, but particularly those professions responsible for direct care of the consequences of this crisis, whether from a medical, psychological or social point of view.

In the area of Social Services, before the pandemic, social workers identified excessive workloads, stress and saturation (39.9%) as the main day-to-day problems, followed by high red tape (16.2%) and the existence of few resources (14.5%) [11]. It can be assumed that due to the COVID-19 these problems have worsened, something that could perhaps have been alleviated in part thanks to the increase in voluntary action [14], with this sector, the volunteers, being precisely the one that has been most overwhelmed according to the results of our study.

In health professionals, the COVID-19 pandemic has generated symptoms of stress, anxiety, depression and insomnia among healthcare workers, with higher levels among women and older professionals [15]. In addition, it has been shown that certain sociodemographic, social and occupational factors significantly increase the risk of suffering problems such as anxiety, depression, stress…: there is a greater risk of suffering these problems depending on sociodemographic factors (greater risk in women and younger professionals), social factors (lack of social support, experiencing social rejection, or stigmatization), and occupational factors (carrying out care tasks on the front line, not having received specialized training on this type of situation, and having less work experience) [16].

As we can see, sociodemographic factors such as being female or being younger, which may cause mental health problems in health professionals, coincide with our results in terms of the groups that have been most affected by the situation. We also found coincidence with respect to occupational factors such as the development of frontline tasks or the lack of training, finding that just over two thirds (69.6%) of the participants have not benefited from training measures, practically 7 out of 10. It is in the private centers where the implementation of these training activities is most perceived, where almost half of the participants (46.3%) declare to have benefited from this measure, as opposed to a low level in the public sector (23.9%).

Although we found a shortage of training activities to address this situation, overall, 52% of the population studied positively values the degree of preparation and effectiveness of the measures implemented by the government compared to 48% of the population that does not value these measures positively. However, with regard to the degree to which the functions carried out in the workplace are affected, it is considerably appreciated that around 84% of people are being affected by the changes produced by the state of alarm, compared to 16% who have not been affected by the situation.

In this respect, a recent survey conducted by Ipsos [17] with almost 26,000 respondents in different countries, placed Spain among the European countries whose inhabitants consider that their government is doing a bad or very bad job with regard to the containment measures implemented (60%, only behind Japan, with 61%). Thus, it seems that the perception of the measures carried out in our country is less negative among Social Services professionals than that indicated by the general population.

The social emergency we are experiencing due to the COVID-19 pandemic highlights the need for an integrated social assistance service [18]. A social assistance system would require (among many things) the collection of vital data that quantifies the effect of COVID-19 on the social assistance sector [19].

The magnitude of this pandemic has confirmed the overall severity of the challenges that society currently confronts. The full impact of the social and economic consequences cannot be predicted at present, but can be intuitively understood from the fact that several countries have implemented total or partial blockades [20].

The WHO [21] has stated that the main psychological shock so far is the high rates of stress and anxiety due to the measures implemented by the government during the state of alarm, the quarantine and the consequences it has brought with respect to the daily routine that was lived before this situation. There has also been an increase in the number of people who find themselves in a situation of loneliness, depression and suicidal behavior. In addition, we must highlight the fear caused by concerns about the effect of the disease on patients with mental health disorders. Ignorance of the impact of the epidemic on people suffering from some kind of mental health disorders will increase the health inequalities that currently exist.

The impact of COVID-19 on mental health, has been reflected in a survey of 5,000 Chinese citizens [20], which shows the following results during the SARS outbreak in 2003: 21.5% registered symptoms of post-traumatic stress disorder, depression (31.2%) symptoms experienced by quarantined citizens surveyed during the SARS outbreak in 2003. According to a survey of 2,091 Chinese citizens [22] the prevalence of PTSD symptoms among the public in mainland China one month after the outbreak of COVID-19 was 4.6%, while the prevalence in high-risk publics, e.g., in the Chinese provinces with the highest number of COVID-19 cases, was 18.4%. In Spain, a study carried out with a sample of 1,933 people [23] revealed that more than a quarter of the participants had reported symptoms of depression (27.5%), anxiety (26.9%) and stress (26.5%) and that, as the period of confinement progressed, the psychological symptoms increased.

Li et al. [24], report a certain increase in the negative emotions discussed above: anxiety, depression and a high sensitivity to social risks. In contrast, there is a large decrease in positive emotions, such as happiness. The frequent use of social networks may be a positive factor in reducing negative mental health effects during the period of the pandemic.

The livelihoods of vulnerable groups in marginal areas are severely affected by the epidemic, and the reduction of income seriously damages the mental health of these groups [25]. In the future, measures must be taken to achieve equity in health to try to prevent mental disorders, depression, stress, anxiety, etc. Providing solutions and care for these patients must be one of the main focuses of health policies and strengthening the health system in Spain to provide emergency responses during the COVID-19 and other public health crises in the future.

Mental health must be a priority for Spain and the rest of the world. The well-being of today's society must be a priority for governments. In the implementation of the proposed containment and de-escalation measures, it is essential and urgent to ensure the mental well-being of people and to try to alleviate the negative effects of the pandemic on the population. Economic and social protection measures that can prevent social inequalities must also be implemented.

## Conclusion

Fear, anxiety, stress, etc. caused by the COVID-19 crisis can affect the well-being of social service professionals. During this pandemic, it is critical that you recognize the signs of stress, take steps to build resilience and manage job stress, and know where to go for help.

Although each person reacts differently to these types of situations, an epidemic like the one we are currently experiencing can generate common traits in terms of mental health. Situations resulting from the state of alarm mean that people experience symptoms derived from social distancing, quarantine or isolation, generating a feeling of anxiety, worry or fear.

However, despite the individual differences, a number of issues can be concluded that have contributed most strongly to the degree to which professionals have been overwhelmed by the situation. In this sense, it can be observed that this fact is positively associated with the degree to which the work of the social services has been affected by the health crisis and the state of alarm, as well as to the perception that these services have been overwhelmed by this situation and the alteration of the usual functions during the crisis period.

The lack of preparation of the service to deal with a situation such as this, as well as the lack of coordination between institutions, the absence of clear and specific instructions with which to act, the fact that it does not have sufficient means to work in a non-presential way and the insufficiency of human resources with which to deal with this situation during the crisis period, have all contributed significantly to generating impotence and overloading of social service professionals who have been overwhelmed by the situation.

### Limitations of the study and proposals for the improvement of social services

One of the limitations of this study, as we have stated in the section "Analysis Strategy", was the difficulty in accessing the sample due to the health emergency and confinement situation in Spain during the time the field work was carried out. Although there was a sufficiently large sample to be able to cover the objectives of the study, obtaining responses from all the regions of Spain, there is an over-representation of some regions over others according to the number of responses obtained.

Another of the limitations we found is that the field work was carried out shortly after the state of alarm was declared in Spain and the different protection measures in the work centres began to be implemented. Therefore, very little time passed from the time the measures were approved until they were submitted for consideration by the professionals. This short period of time may be a factor that influences the assessment made, so it would be interesting to continue to deepen this line of work, complementing the results presented here with future research that considers the evolution of the measures as well as the perception of professionals over time.

However, we believe that the work is particularly interesting to evaluate the perception about the safety conditions and the measures carried out in the different workplaces belonging to the Social Services system in Spain, so that it can serve for a better preparation in case of having to go through a similar situation and prevent possible difficulties detected during the process. This is therefore an initial opinion of professionals who at that time were immersed in a situation in which their working and personal conditions were being drastically modified by the pandemic, and the experience gathered in this work could serve to improve services in the future.

In this way, this situation can become a real opportunity to improve the quality and efficiency of services, for example, it could be interesting to think about how greater coordination could be facilitated with other systems, especially the health system, or to go deeper into teleworking and how to improve conditions for it in order to take advantage of the experience of telematic work carried out during confinement and explore whether continuing to provide some services through this route would mean speeding up the system. It also represents an opportunity to reflect on the digital divide that affects many users of Spanish social services. How to overcome this gap is one more challenge that the Spanish social services system must face.

Finally, the questionnaire included open-ended questions in which the participating professionals found a space to vent their concerns not only at work, but also personally and with their families, and the qualitative analysis of these issues that the research team is working on could also provide very interesting information from the point of view of mental health and occupational health, with a view to the possibility of implementing mechanisms in social services to prevent or mitigate related problems such as anxiety, depression, stress or burnout.

## Supporting information

S1 File Analysis data in SPSS format have been made available to the journal. (ZIP)

S1 Appendix Full questionnaire used in the research translated into English^1^. (DOCX)
