# Supplementary material for: COVID-19 and social services in Spain
Source: PLoS One. 2020 Nov 18;15(11):e0241538. doi: 10.1371/journal.pone.0241538 (PMC7725485; doi:10.1371/journal.pone.0241538)
Supplement: S1 Appendix — (DOCX) [file pone.0241538.s002.docx]

**S1 Appendix. Full questionnaire used in the research translated into English^[[1]](#footnote-1)^.**

| **Sections of the questionnaire** | **Included questions** |
| --- | --- |
| 1. Socio-demographic characteristics and the professional situation | 1.1. Sex or gender  1.2. Age  1.3. Civil Status  1.4. Do you have children?  1.5. If you answered "Yes" in the previous question, how many children live with you?  1.6. Educational level (please indicate the highest)  1.7. If you have a university degree, please indicate which one  1.8. Employment situation  1.9. Time in years you have worked in your current entity/organization/service  1.10. Type of organisation you work for  1.11. Indicate the main group with which you carry out your professional activity (social exclusion, people with disabilities, migrants, gender violence, minors, the elderly, etc. or generalist service - it covers all groups).  1.12. In which town do you work?  1.13. What position do you hold in your workplace? |
| 2. Assessment of the impact of Covid-19 and the state of alert on the development of social services  (16 items)  *They score from 1 (strongly disagree) to 5 (strongly agree)* | 2.1. The work we carry out from the social services has been greatly affected by the appearance of covid-19 and the state of alarm.  2.2. I think that in the service I work in we were sufficiently prepared for a situation like this.  2.3. The response offered by the social services as a system has been adequate to the situation created.  2.4. The population using social services is aware of the measures put in place during the state of alert.  2.5. The population that uses social services appreciates the measures put in place in a positive way.  2.6. Coordination between institutions to organise the response of social services as a system to covid-19 has been satisfactory.  2.7. As professionals, we have had clear and concrete instructions on how to act in this situation.  2.8. In my job, teleworking has allowed me to carry out my professional work normally.  2.9. I have had sufficient means to telework during the development of the crisis.  2.10. I have had the necessary training and instructions to be able to carry out my work telematically or not.  2.11. The declaration of social service professionals as essential seems to me to be right.  2.12. In general, it can be said that the social services system is overwhelmed by this situation.  2.13. The available human resources are sufficient to develop our services during the state of alarm.  2.14. My usual duties have been altered during the crisis period.  2.15. The implementation of the new measures derived from the state of alarm has been done effectively and efficiently.  2.16. Please tell us about any experience and/or professional situation that has caught your attention or impacted you during this time. Or point out one or more alternative proposals to the measures developed. Also assess your perception of the future of social services (open-ended question) |
| 3. Indicate whether or not you are aware of the protection and information actions carried out in the workplaces  (8 items)  *Yes or no answers* | 3.1. Messages or circulars have been prepared and posters have been placed at the entrance, indicating to visitors that they should not go to these centres unless it is essential and in no case if they present any respiratory symptoms or fever.  3.2. Information posters on hand hygiene and respiratory hygiene have been placed at the entrance to the centres, corridors and common areas.  3.3. Training activities have been carried out for the health education of users and workers on hand and respiratory hygiene.  3.4. It has been ensured that all toilets have soap and paper towels for hand hygiene.  3.5. There are dispensers with hydroalcoholic solution for hand hygiene, disposable tissues for respiratory hygiene and waste containers, with a lid that opens with a pedal.  3.6. Measures have been taken to minimise interaction with users and other professionals  3.7. Intensified cleaning programmes have been implemented in the centre, with special attention to areas where the greatest number of people may transit and to surfaces of frequent contact such as railings and handrails, buttons, door knobs, tables, etc.  3.8. Workers and users of the centre have been informed of the actions being taken to protect them. |
| 4. Assess your personal professional situation during the alarm state  (13 items)  *They score from 1 (strongly disagree) to 5 (strongly agree)* | 4.1. I have often felt like crying these days.  4.2. Throughout these days, discussions with social service colleagues have increased.  4.3. I have often felt support and understanding from users of social services for the difficulty of the situation.  4.4. During these weeks, I have worked many more hours beyond my working hours.  4.5. It can be said that there have been times when I have felt overwhelmed by the situation.  4.6. I have had the necessary protective equipment to do my job.  4.7. My professional mobility has not been affected. I have been able to travel to my workplace without any problems.  4.8. I have had the basic training to face my work during this time.  4.9. At my workplace I have been properly accredited to do my job during the alarm period.  4.10. In general, I have found support from my colleagues in solving the problems I have faced these days.  4.11. I have often felt helpless these days.  4.12. I'm teleworking at home and going to work one day.  4.13. Despite teleworking, I know that at any time I can be called back to work |
| 5. Assessment of actions implemented for the protection of vulnerable groups  (24 items)  *They score from 1 (strong negative perception) to 5 (strong positive perception)* | 5.1. The strengthening and reorganisation of existing social resources by the municipal social services has been adequate.  5.2. The municipality where I live has made an accurate diagnosis of the situation in which the most vulnerable populations find themselves during the health crisis.  5.3. The collaboration with third sector entities is being fundamental to attend to these groups.  5.4. The measures taken to ensure the confinement of vulnerable populations and segregated settlements have achieved their objective.  5.5. Volunteers are doing a great job right now to help people in vulnerable situations.  5.6. The measures taken for the socio-health monitoring of this population have allowed adequate attention to be given to it.  5.7. The measures taken (financial or material assistance, processing of minimum income, extraordinary benefits...) to guarantee the income of these families are being managed rapidly.  5.8. I believe that there are unmet vital needs.  5.9. With regard to cash benefits, the creation of a fixed cash advance to ensure the immediate delivery of aid is working well.  5.10. The systems implemented to replace the canteen grants and guarantee adequate food for the children are proving effective.  5.11. The measures that have been developed to cater for early childhood (0-3 years) and pregnant women are appropriate.  5.12. Programmes to monitor children's homework and social vulnerability are ensuring that their school performance is not delayed.  5.13. In the service in which I develop my work, there is an effective coordination that allows to raise the knowledge of the professionals directly involved in the attention to the vulnerable population and higher levels of decision making.  5.14. The paralysis of the social intervention projects (accompaniment) in relation to the social and labour inclusion, is causing a stop in the access to potential jobs of the vulnerable population.  5.15. The paralysis of the administrative procedures for access to the Minimum Income of Insertion is aggravating the living conditions of the most vulnerable population.  5.16. The suspension of Day Care Centres and Home Help Services that are not considered Minimum Services creates a problem of work-life balance for carers of dependent people and an overload of their care tasks (work responsibilities, care of children and the elderly).  5.17. Confinement has severely affected people with mental health problems and mental illness and their families.  5.18. Confinement is seriously affecting women victims of gender-based violence and their children.  5.19. Most social service users do not have access to telematic procedures, and telephone assistance is sometimes not sufficient.  5.20. The state of alarm and the health alert has made people vulnerable who, until now, were only in precarious conditions.  5.21. In general, I believe that once the state of alarm and the health alert is over, the living conditions of the vulnerable population will have worsened.  5.22. Reflecting on the future of the vulnerable population, which cases or profiles will be most affected and why? (open-ended question)  5.23. What alternatives or measures do you consider have not been taken into account in the measures implemented by the competent governments and which are necessary for the care of vulnerable groups? (open-ended question)  5.24. Tell us about an experience or situation that has caught your attention during this time (open-ended question) |

1. The complete questionnaire in Spanish that was carried out online can be found at: <https://forms.gle/DgrieGyGHbsECM6k9> [↑](#footnote-ref-1)
